# Burden of the family caregivers of the person with mental illness in portugal: A cross sectional study

**DOI:** 10.1192/j.eurpsy.2021.1035

**Published:** 2021-08-13

**Authors:** C. Laranjeira, A. Querido

**Affiliations:** Citechcare, Polytechnic of Leiria, Leiria, Portugal

**Keywords:** Caregiver Exaustion, Family Caregivers, Person with Mental Illness, Situational Transition

## Abstract

**Introduction:**

The provision of mental health care should be promoted at the community level, in order to facilitate their recovery process. Thus, the people who care for these individuals go through a situational transition, as they have to play the role of Family Caregivers (FC).

**Objectives:**

a) to characterize the FC burden of the person with Mental Illness; and b) to correlate FC overload with the variables age, gender, cohabitation, degree of kinship and level of education.

**Methods:**

A cross-sectional correlational study was conducted. The sample consisted of FC who went to consultations and to the inpatient psychiatric unit of a portuguese hospital. Data were collected through a questionnaire which included sociodemographic data, the Lawton-Brody Index and the Zarit Burden Interview (ZBI).

**Results:**

Of the 119 FC, 66.4% were female, with an average age of 53.8 years. 73.1% of FC cohabited with the person they care for, in which the majority was cared for by their child or spouse, 45.2% and 36.1%, respectively. It should also be noted that 71.4% felt the need for more support from health professionals. As for autonomy, 52% of the individuals cared for by FC were moderately dependent. It was verified that about 45% of the FC had an intense overload. Female gender has greater overload and that there are no significant differences between the level of education and cohabitation.

**Conclusions:**

Considering the results, it is understood the importance of valuing FC, as a target and care partners, in order to reduce the burden, they feel when caring for people with MI.

